# Progress of biological agents and CAR cell therapy in the treatment of common autoimmune diseases in children

**DOI:** 10.3389/fped.2026.1742663

**Published:** 2026-02-26

**Authors:** Qi-Ling Yin, You-Qiong Liu, Hui-Min Zhang, Yu-Dong Gao, Ying Wang, Wei-Hua Zhang

**Affiliations:** 1 First Clinical Medical College, Shaanxi University of Chinese Medicine, Xianyang, Shaanxi, China; 2 Department of Pediatric Respiratory, Rainbow Hospital of Xianyang (Children’s Hospital of Xianyang), Xianyang, Shaanxi, China

**Keywords:** autoimmune diseases, biological agents, CAR cell therapy, children, immunomodulatory agents

## Abstract

Autoimmune diseases are characterized by immune disorders that lead to abnormal activation of autoreactive immune cells, which in turn lead to tissue destruction and organ dysfunction. Compared with adults, autoimmune diseases in children are more severe, with increased disease activity and organ damage occurring earlier, and with higher mortality. In addition to the poor effect of traditional therapy in some children, children also have the needs for growth and development, and the use of traditional therapy will lead to severe immunosuppression and sequelae, affecting the quality of life of children. The emergence of Chimeric antigen receptors (CAR) cell therapy and biological agents has provided new treatment options for children with autoimmune diseases.

## 1 Introduction

Autoimmune diseases are a group of diseases characterized by an abnormal immune response of overactivated T cells and B cells to normal components of the body. These diseases can affect any tissue and organ and occur in all age groups (1–3). Autoimmune diseases have been shown to affect 3%–5% of the population and become one of the most important public health problems (4). In pediatric autoimmune diseases, The most common diseases include juvenile idiopathic arthritis (JIA) and juvenile systemic lupus erythematosus (SLE) erythematosus (JSLE), juvenile-onset systemic sclerosis (JSSc), etc (5). Compared with adults, autoimmune diseases are often more severe in children. For example, JSLE generally presents with a more aggressive phenotype, characterized by increased disease activity and earlier onset of organ damage, and frequently causes lupus nephritis (LN) and neurologic involvement, with a higher mortality rate than adult-onset SLE (6). The treatment of autoimmune diseases in adolescents is particularly challenging. The traditional treatment is mainly to use systemic glucocorticoids (GC) or broad-spectrum immunosuppressants to reduce systemic hyperimmunity. Although this traditional therapy can relieve symptoms, it often leads to severe immunosuppression, the development of serious complications, and an increased risk of infection and organ damage (7–9). Therefore, in order to achieve the best control of the disease, improve the quality of life of children and reduce the occurrence of sequelae, it is very important to explore new treatment methods.

## 2 Literature search strategy

### 2.1 Search databases

The PubMed database was utilized for this review.

### 2.2 Search keywords

Children, Autoimmune diseases, Biological agents, CAR cell therapy, Chimeric antigen receptor, Juvenile idiopathic arthritis, Juvenile systemic lupus erythematosus, Juvenile-onset systemic sclerosis, Juvenile dermatomyositis.

### 2.3 Search time period

The search was limited to the period from January 2015 to October 2025.

### 2.4 Literature screening criteria

Inclusion criteria (1): study type: randomized controlled trials, cohort studies, case-control studies, case reports, systematic reviews, and meta-analyses; (2) study population: patients with autoimmune diseases aged ≤18 years, or studies including subgroup analyses of adults and children; (3) research content involving therapeutic efficacy, safety, or mechanisms of action of biologics or CAR cell therapies in autoimmune diseases.

Exclusion criteria: (1) duplicate publications; (2) low-quality literature with incomplete data or inability to extract valid information; (3) original studies cited in review articles that were independently included.

## 3 New advances in biological agents

### 3.1 Anti-CD20 monoclonal antibody: rituximab (RTX)

CD20 is a transmembrane calcium channel implicated in B-cell activation, proliferation, and differentiation, and it is present on the surface of B cells at stages ranging from late pre-B cells to mature memory B cells. RTX is a chimeric mouse/human monoclonal antibody that binds to CD20, which is expressed on the surface of pre-B cells and mature B lymphocytes, leading to the apoptosis of these cells through antibody-dependent and complement-dependent cytotoxic effects. The clinical benefit of B-cell depletion may result from the loss of other significant B-cell functions, such as antigen presentation, inflammatory cytokine production, T-cell activation, and ectopic lymphoid follicle formation. Based on this theory, the use of this class of drugs in other autoimmune diseases with both T cell and B cell etiology, such as SLE (10, 11).

JSLE is the second most common rheumatic disease in children after JIA. However, the use of GCs to control disease activity in children may result in a variety of adverse effects. Because B cells play a central role in SLE pathophysiology, modulating memory or autoreactive B-cell lines and working to reestablish a balanced B-cell repertoire is a logical approach to controlling the disease and achieving improved disease control, remission, and ongoing stability. In JSLE, B-lymphocyte stimulator levels are elevated, leading to decreased self-tolerance, and RTX is a chimeric monoclonal antibody that depletes B cells by targeting CD20 (12, 13). In patients with JSLE, the use of RTX significantly improved disease activity, markers of serum and urinary disease activity, and reduced oral corticosteroid doses; However, hypogammaglobulinemia occurs in about 13% of patients. Therefore, monitoring IgG levels before RTX treatment is critical and should be used with caution in patients with pre-existing hypogammaglobulinemia (14). The SHARE group also recommends the use of RTX in combination with another disease-modifying antirheumatic drugs (DMARDs) in patients with refractory LN (15). New treatments for CD20 in SLE. Atacicept and telitacicept are anti-CD20 monoclonal antibody drugs. It can inhibit B-lymphocyte Activating Factor of the tumor necrosis factor Family (BAFF) and A PRoliferation-Inducing Ligand (APRIL), BAFF and APRIL are important B cell regulatory cytokines. Therefore, treatment regimens targeting BAFF and APRIL seem to be promising drug options for SLE patients, but relevant clinical data are currently lacking (12).

B-cell antigen-driven autoimmunity in Idiopathic Inflammatory Myopathies (IIM) is characterized by clonal expansion, class-switching somatic mutations, and plasma cell maturation. In addition, B cells also act as antigen-presenting cells that costimulate *T* cells and secrete proinflammatory cytokines, making them a reasonable target for adult dermatomyositis (DM) and juvenile dermatomyositis (JDM) (16). The results of the first trial of RTX in myositis showed that although the study did not provide sufficient evidence to reject the null hypothesis of no treatment effect in the primary and secondary outcomes, the trial data showed that the addition of RTX significantly reduced GC use and that approximately 83% of enrolled patients achieved disease improvement targets. Of note, these patients belong to a group of patients with refractory myositis that does not respond to treatment with GC and who have on average used more than three other immunosuppressants during the course of their disease (17).

In a small observational study, Zulian et al. used RTX in combination with mycophenolate mofetil to treat four patients with rapidly progressive JSSc. After treatment, all patients showed a reduction in the frequency and duration of Raynaud's phenomenon, a reduction in skin involvement, and a reduction in the juvenile systemic sclerosis severity score (J4S). In addition, cardiac function improved in two patients and respiratory function improved in the remaining two patients, both without serious adverse effects (18).

### 3.2 Anti-tumor necrosis factor (TNF-α) antagonists

#### 3.2.1 Infliximab (IFX)

TNF-α levels are higher in JDM patients with a long course of disease and calcification, which is a complication that may lead to severe impairment of physical function, and there is some evidence that long-term active disease is related to this complication, and its incidence can be reduced by early control of the disease. Therefore, TNF may be a good potential therapeutic target for the treatment of JDM. Studies have shown that symptoms of muscle and skin disorders are reduced after treatment with IFX and Adalimumab(ADA), IFX and ADA are well tolerated, and most patients do not experience adverse effects (19).

#### 3.2.2 Etanercept (ETA)

In 2014, two trials of ETA in the treatment of JDM showed that ETA did not show significant or reliable improvement in the control of JDM. Although beneficial for some patients, clinical data are limited, and caution is still needed when using ETA for JDM (20, 21). Although TNF inhibitors, especially ADA or IFX, may be helpful in some patients with JIIM, evidence from systematic reviews suggests that treatment with these drugs does not lead to complete remission and that better treatment is needed (22).

### 3.3 IL-6 receptor antagonist: tocilizumab (TCZ)

Studies have shown that the serum concentration of IL-6 in patients with systemic sclerosis (SSc) is higher than that in healthy controls, and IL-6 may be involved in the pathogenesis of early SSc. However, TCZ is an IgG antibody capable of binding to the IL-6 receptor, which acts by inhibiting the binding of IL-6 to the IL-6 receptor (23). Current approved pediatric indications for TCZ include systemic and polyarticular JIA. In a retrospective 2020 study of 30 patients with JSSc, 9 patients with pulmonary and/or gastrointestinal involvement who had been treated with conventional therapies such as steroids and DMARDs were treated with TCZ. The modified Rodnan skin score (mRSS) and J4S decreased in all patients, and the chest high-resolution CT improved in all patients. All patients tolerated TCZ well. TCZ is a potential treatment option for JSSc patients who do not respond to conventional treatment regimens, but long-term prospective studies with more patients are still needed to provide more relevant data (24).

### 3.4 *T* cell inhibitor: abatacept (ABA)

CD28 is a key mechanism in autoimmune pathogenesis, and ABA is a recombinant fusion protein that inhibits *T* cell activation by binding CD80/86 and preventing costimulatory signaling by CD28 (25).

In the study by De Guzman et al., four JDM patients were treated with ABA to relieve calcinosis, two of whom had previously been treated with RTX. ABA was administered at a dose of 10 mg/kg at weeks 0, 2, 4, and every 4 weeks thereafter. All patients showed improvement in clinical symptoms and imaging findings, as well as alleviation of pain, tenderness, and inflammatory changes at the site of calcium deposition. More importantly, no serious side effects were reported during treatment (26). ABA has demonstrated efficacy in randomized controlled trials of adult myositis 179 and in open-label treatment trials of JIIM. ABA may be useful in the treatment of resistant diseases, including calcinosis (22).

### 3.5 Janus kinase (JAK) inhibitors

A growing body of evidence suggests that type I and type II interferons (IFN) play a role in JDM and DM, including elevated IFN-responsive gene signatures in muscle, skin, and blood. Clinically, up to 80% of myopathic DM patients can develop interstitial lung disease (ILD), and if there is MDA5 autoantibodies, it is more associated with ILD (up to 95%). ILD is a complication of high concern in MDA5 autoantibody positive JDM because of its refractory course and high mortality (up to 50% at 6 months). There is emerging evidence that JAK inhibitors benefit patients with JDM by reducing type I IFN signaling. Given the critical role of the JAK STAT pathway in disease pathophysiology, JAK inhibitors are a reasonable therapeutic option for JDM. Currently reported JAK inhibitors for the treatment of JDM include baricitinib, tofacitinib and ruxolitinib. Among them, ruxolitinib and baricitinib only inhibit JAK 1/2. tofacitinib inhibits one third of Jacs (27–30).

#### 3.5.1 Baricitinib

The case of an 11-year-old boy with refractory JDM treated with baricitinib was reported in 2019, This case showed that the patient had been treated with azathioprine, mycophenolate mofetil, IFX, ADA, RTX, tacrolimus, cyclosporine, intravenous immunoglobulin (IVIG) sequentially over the past 7 years. However, the patient still had persistent severe skin disease activity and progressive calcinosis. After the child was treated with baricitinib, the symptoms improved significantly. Biomarkers of IFN signaling, type I IFN-induced gene expression, and STAT1 phosphorylation in CD4+, CD8+, and CD14+ cells were all reduced to levels comparable to healthy controls. Meanwhile, circulating endothelial cells, a biomarker of endothelial damage associated with JDM vasculopathy, were also significantly reduced after treatment. Of note, this patient achieved a significant reduction in corticosteroid dose for the first time after treatment with baricitinib, halting the progression of calcinosis and overall improvement (31). In the first comprehensive prospective evaluation of JAK inhibitors (baricitinib) for JDM in 2022, four JDM patients with chronic active disease and suboptimal response to 3–6 immunomodulatory drugs were treated with baricitinib for 24 weeks. All patients had significant improvement in clinical scores and reduction in IFN markers. No serious adverse events occurred during this period, and no subject discontinued baricitinib (32). In a retrospective study involving 20 patients with refractory JDM, rash improved in 95% of patients after treatment with baricitinib, and all patients with myasthenia at enrollment improved by week 24 (33).

#### 3.5.2 Tofacitinib

Tofacitinib inhibits the signaling of type I and type II cytokine receptors that bind to Tyk2, JAK1, JAK 2, and JAK 3 and blocks the signaling of IL-2, IL-4, IL-15, IL-21, IFN-γ, and IL-6. It also blocked IL-12 and IL-23 signaling to a lesser extent. As a result of this broader activity, tofacitinib impairs the differentiation of CD4+ T helper cells (Th1 and Th2) and limits the production of pathogenic Th17 cells. In a 2019 study, two anti-MDA5 autoantibody positive patients with refractory JDM were treated with RTX and ABA, but the response was not satisfactory. After tofacitinib treatment, both patients had improvement in disease activity, and the results showed that STAT1 phosphorylation in CD4+ *T* cells and monocytes stimulated with a variety of cytokines improved to levels comparable to healthy controls. This highlights the broader immunomodulatory effects of tofacitinib than inhibition of type I IFN. One such patient, who had a history of recurrent mucositis and was cured with acyclovir, developed herpetic meningitis during treatment with tofacitinib (29). In a 2023 retrospective study of 88 patients with refractory JDM treated with tofacitinib for > 3 months, nearly 50% achieved complete clinical response; Six patients were treated with tofacitinib monotherapy, 60% of patients with interstitial lung disease recovered well on high-resolution CT, 75% showed a reduction in the size or number of calcinosis, and 25% showed complete resolution of calcinosis. Of note, the study did not find any serious adverse effects; Only one patient developed a herpes zoster infection after 9 months of treatment. After drug discontinuation, herpes resolved and tofacitinib treatment was resumed (34).

One study reported that a 13-year-old girl with JSSc, unresponsive to treatment with corticosteroids, mycophenolate mofetil, and RTX, showed marked clinical improvement and normalization of inflammatory markers after 1 month of treatment with tofacitinib (35).

#### 3.5.3 Ruxolitinib

In 2023, ruxolitinib was selected for a 4-year-old MDA5-antigen-positive child with JDM and ILD who had a poor response to first-line and ADA therapy and a markedly elevated IFN profile. After treatment with ruxolitinib, the patient's condition continued to improve, with chest CT showing a near complete response, and treatment with oral GC was tapered and eventually discontinued after 6 months, as was IVIG after 13 months. Several studies suggest that extended treatment with JAK inhibitors may be a useful alternative to other immunosuppressive approaches such as RTX or cyclophosphamide (30). At present, the role of JAK inhibitors in JDM has not been definitively determined. Since JAK inhibitors are not first-line recommended drugs and may cause side effects, individual clinical considerations should be taken into account when treating JDM.

### 3.6 Recombinant human monoclonal antibody against BAFF: belimumab

BEL is a human monoclonal antibody that targets BAFF. The level of soluble BAFF in SLE patients was significantly higher than that in healthy individuals. BAFF levels are also increased in the active stage of disease. BAFF is a key regulator of B cell survival, differentiation and maturation, which can bind to receptors on B cells, thereby inhibiting B cell apoptosis and promoting proliferation and differentiation. In 2019, the FDA approved intravenous BEL for use in patients 5 years of age or older with JSLE. In SLE patients, B cell activation caused by various factors is the main pathogenesis. BEL can bind and antagonize soluble BAFF, block its interaction with B cell receptors, reduce the survival rate of B cells, induce the apoptosis of autoimmune B lymphocytes, and thus reduce the production of autoantibodies (36–38). A 2020 trial of intravenous BEL for active JSLE, the PLUTO study, evaluated intravenous BEL (10 mg/kg) plus standard JSLE therapy vs. placebo in 93 patients with JSLE. The proportion of patients who achieved the main efficacy index of SLE response index was higher in patients treated with BEL. The BEL intravenous pharmacokinetic profile and the benefit-risk profile of JSLE were consistent with those of the BEL study in adults. It was also confirmed that intravenous 10 mg/kg is appropriate for the pediatric population (39). In a retrospective study on JSLE in 2022, 49 children were divided into the BEL treatment group (18 cases) and the traditional treatment group (31 cases). The traditional treatment group was treated with prednisone and traditional immunosuppressive agents, and the BEL group was treated with BEL in addition to the traditional treatment. The results showed that the oral prednisone dose in the BEL treatment group was lower than that in the conventional treatment group. At 24 weeks of treatment, the prednisone dose and incidence of adverse events were lower in the BEL treatment group than in the conventional treatment group (40).

## 4 CAR cell therapy

CAR are genetically engineered receptor molecules that endow immune effector cells with the ability to recognize specific antigens. CAR platforms are diverse, with various cell types, such as CAR T, CAR NK, and CAR M, and may be suitable for different disease Settings. Among them, CAR T cell therapy has achieved remarkable success in the treatment of hematological malignancies, and a number of products have been approved by regulatory authorities. CAR NK cells have anti-tumor activity and immunomodulatory properties. The efficacy and safety of CAR NK cells in hematological malignancies were initially verified in a clinical study conducted in 2020. CAR M cell therapy, characterized by its superior tumor infiltration capacity, tumor cell phagocytosis, antigen presentation, and inflammatory cytokine secretion, has been proposed as a novel strategy to overcome the immunosuppressive nature of solid tumors (41). The efficacy of CAR *T* cells is best demonstrated by the successful use of CD19-targeting CAR *T* cells in B-cell non-Hodgkin's lymphoma in adults and B-cell acute lymphoblastic leukemia in children and adults, with complete remissions in up to 85% of patients treated with a single dose. Anti-cd19 CAR T cells can cause abnormal B cell development. Theoretically, this therapy could be used to treat B-cell-mediated rheumatic diseases, including SLE, IIM, SSc, and other B-cell-mediated autoimmune diseases (42). Direct cell-cell contact with cytotoxic interactions by CAR *T* cells can result in more robust and sustained responses than conventional therapies, with the ability to invade tissues such as the skin, kidney, and even bone marrow and lymph nodes, leading to more complete depletion of B cells in a variety of tissues (43).

### 4.1 JSLE

Compared with SLE, JSLE has a more active course and LN is more common, and the presence of LN is associated with increased mortality. Studies have shown that only 40%–60% of JSLE patients achieve the treatment goal of complete LN remission with conventional therapies, and about 62% of JSLE develop organ damage and impaired quality of life in adulthood (44, 45). In 2024, CAR *T* cell therapy was first used for the treatment of severe and rapidly progressive LN in children. After multi-line treatment with high-dose methylprednisolone, cyclophosphamide, hydroxychloroquine, azathioprine, mycophenolate mofetil and BEL, the patient's condition continued to deteriorate, and finally the patient required hemodialysis and antihypertensive drugs. In contrast, after the administration of CD19-targeting CAR T cells, disease activity rapidly decreased, renal function improved, and sustained clinical and laboratory responses were achieved with the discontinuation of hemodialysis 17 days after CAR T-cell therapy (58). In 2025, two patients with refractory JSLE were reported to have been successfully treated with CD19-targeting CAR *T* cells. They both developed renal-related impairment following various immunosuppressive therapies prior to CAR T therapy, but showed significant clinical and laboratory improvement after autologous CD19-targeting CAR T cell therapy. GC and other immunosuppressants were stopped at the same time, which showed good tolerance and significant efficacy in late follow-up (59).

CAR NK cells have shown a clear safety advantage over CAR *T* cells, with minimal serious side effects reported in early clinical trials. In addition, sources such as cord blood, stem cells, and cell lines can be used to generate CAR NK cells, not only reducing manufacturing costs but also improving clinical feasibility. Taking advantage of these advantages, investigators have begun to investigate CAR NK therapy as a treatment option for SLE. Currently, in an *in vitro* study and in a lupus-like humanized mouse model, PD-L1-CAR NK cells selectively eliminated PD-1-high CD4+ *T* cells, including follicular helper *T* cells, resulting in reduced memory B cell proliferation, differentiation, and Ig antibody secretion (41).

In the existing studies, the successful treatment of JSLE with CAR T cells involves only a few case reports of patients aged 12–15 years, which is not only relatively limited in age group, but also small in sample size. More data on younger age groups are needed to further confirm the safety and efficacy of CAR T cell therapy. Whether CAR NK cell therapy can be used in the treatment of JSLE remains to be confirmed.

### 4.2 JDM

JDM is the most common type of IIM in childhood, and its etiology is still unclear. The current treatment is GC combined with methotrexate, but due to the unsatisfactory efficacy, most patients require other immunomodulatory therapy, and the chronic disease activity, damage and mortality are still unacceptable. The first use of CAR T-cell therapy for JDM occurred in 2024, and CD19-targeting CAR T cells were shown to be an effective treatment option for refractory JDM. The JDM patients in this study had chronic active disease with severe muscle and skin involvement that responded only transiently to repeated immunosuppressive therapy. In contrast, the use of CD19-targeting CAR T-cell therapy can achieve complete depletion of B cells in the peripheral blood while avoiding the side effects associated with long-term immunosuppression in patients with autoimmune diseases due to autoreactive B cells (46).

### 4.3 SSc

In the majority of SSc patients, early initiation of treatment can control disease progression, resulting in a good quality of life. However, in some cases, JSSc may progress rapidly or be unresponsive to treatment. In some cases, the disease can be life-threatening. In 2024, four adult patients with refractory SSc were reported to have complete resolution of disease symptoms and reduced severity of skin and lung disease after therapy with CD19-targeting CAR T cells, demonstrating the ability to stabilize or even improve fibrotic manifestations while also rapidly improving SSc inflammatory manifestations, including arthritis and myositis. More notably, all patients were successfully weaned from immunosuppressive drugs without relapse or disease worsening (47, 48). At present, there are only a few case reports of CAR T cell therapy in adult SSc, and there is no related report in JSSc, so whether CAR T cell therapy can be used in JSSc needs to be further studied.

Few cases of CAR T-cell therapy have been reported in Sjogren's syndrome, IIM, rheumatoid arthritis, and immune thrombocytopenia, but all have been limited to adult patients, and all require further data to confirm their safety and efficacy (41, 49).

### 4.4 Side effects of CAR cell therapy

Although CAR cell therapy shows promising applications in treating common autoimmune diseases in children, it also carries certain side effects that require close monitoring and prompt management during clinical use. Currently, CAR T-cell therapy, as the primary form of CAR cell therapy, is more widely applied clinically. Consequently, research on the side effects of such therapies has primarily focused on CAR *T*-cell therapy. Existing research indicates that CAR *T*-cell therapy primarily involves two toxic reactions: cytokine release syndrome (CRS) and immune effector cell-associated neurotoxicity syndrome (ICANS), with CRS being the most common (50, 51).

CRS is a systemic inflammatory response characterized by fever, chills, tachycardia, cellular edema, hypotension, and hypoxia, accompanied by elevated inflammatory biomarkers such as C-reactive protein, ferritin, and interleukin-6 (IL-6). It typically occurs within the first week post-infusion. Its pathogenesis involves activated CAR-*T* cells releasing cytokines such as IFN-γ, TNF-α, and granulocyte-macrophage colony-stimulating factor. These factors stimulate myeloid cells, inducing massive release of cytokines including IL-1β, IL-6, and TNF-α, ultimately leading to cytopenias and cardiovascular or respiratory symptoms. CRS can be managed with antipyretics, corticosteroids, and first-line therapeutic agents like TCZ; if symptoms persist, the interleukin-1 receptor antagonist anakinra may be considered. ICANS primarily occurs in patients receiving CAR *T*-cell therapy for malignancies and has not been reported in autoimmune disease treatment (52).

Compared to the application of CAR T-cell therapy in hematologic malignancies, the incidence of CRS is lower in patients with autoimmune diseases, and no cases of ICANS have been reported. However, it is noteworthy that a distinct category of localized adverse reactions, termed local immune effector cell-associated toxicity syndrome (LICATS), has been observed in SLE, SSc, and IIM patients receiving CD19 CAR T-cell therapy. These symptoms are typically mild and resolve spontaneously or following short-term GC treatment (53, 54).

Additionally, studies indicate that some patients with autoimmune diseases may develop infections, primarily mild upper respiratory tract infections, during the later stages of CAR T-cell therapy. Others may experience hypogammaglobulinemia, with a small proportion requiring IgG replacement therapy (55).

## 5 Discussion and conclusions

As we all know, with the in-depth research on biological agents, they can not only be used for the treatment of Kawasaki disease, but also gradually expand the scope of application in children with autoimmune diseases. On the basis of the current study, biologics for the treatment of JSLE in addition to first-line therapy include RTX in an anti-CD20 monoclonal antibody and BEL, a recombinant human monoclonal antibody against BAFF. Because there are relatively many studies on RTX in the treatment of JSLE, for the choice of drugs, RTX can be considered when the first-line treatment is not effective. Since RTX may cause hypogammaglobulinemia in a small number of patients, it is necessary to monitor IgG levels before and after treatment with RTX.

At present, the biological agents used for the treatment of JDM include RTX in anti-CD20 monoclonal antibody, IFX and ETA in anti-Tnf-α, ABA in T cell inhibitors, and baricitinib, tofacitini and ruxolitinib in JAK inhibitors. Among them, anti-Tnf-α agents are the most common type of biologic agents in JDM, which have improved both cutaneous vasculitis and active myositis, and in treated patients, the use of steroids is reduced. ETA showed reduced efficacy on muscle inflammation, with data indicating that only 16% of treated patients achieved complete clinical remission. Since calcinosis is a serious complication of JDM that causes severe functional impairment and there is no definitive treatment, it may be useful to treat patients with calcinosis with IFX or ADA. Anti-tnf-α agents may reduce preexisting lesions or prevent the development of new lesions (56). To date, the role of JAK inhibitors in JDM has not been finally determined, and JAK inhibitors are not recommended drugs. The current studies are used when other biological agents are not effective, so the clinical condition of patients and the side effects of drugs need to be considered comprehensively. In JSSc patients, RTX is the most commonly used biological agent, followed by TCZ. Among them, RTX is mainly used to treat cardiac involvement, while TCZ is more inclined to treat pulmonary involvement (57).

CAR T-cell therapy can be used to treat JSLE, JDM, and adult SSc; no data are available to support its use in JSSc. CAR *T*-cell therapy can lead to more robust and sustained responses through direct cell-cell contact with cytotoxic interactions than conventional therapies. However, due to the economic burden and the relative limitations of current research, CAR *T* cell therapy is usually not the first choice for children with refractory autoimmune diseases.Although side effects from CAR *T*-cell therapy are generally milder in patients with autoimmune diseases compared to those with hematologic malignancies, close monitoring and prompt intervention remain essential both before and after treatment.

This study has certain limitations. Currently, the use of biologics and CAR cell therapies for treating pediatric autoimmune diseases remains in the clinical exploration phase. Relevant clinical research data is insufficient, with most studies being small-sample, single-center retrospective investigations or case reports. Large-scale, multicenter, long-term prospective randomized controlled trials are still lacking. Additionally, the long-term impact of both biologics and CAR cell therapies on pediatric patients’ outcomes—such as disease recurrence rates, organ function preservation, and quality of life—remains unclear, necessitating long-term follow-up studies to supplement relevant data.

In summary, traditional therapies for autoimmune diseases usually lead to severe immunosuppression, and some children are not sensitive to traditional therapies. Therefore, the emergence of CAR cell therapy and new biological agents provides new treatment options for children. However, at present, there are only a few case reports on CAR cell therapy and emerging biological agents for the treatment of children with autoimmune diseases, and more clinical data are needed to further confirm their efficacy and safety.
